# Huisheng Oral Solution Adjunct With Platinum-Based Chemotherapy for the Treatment of Advanced Non–Small Cell Lung Cancer: A Meta-Analysis and Systematic Review

**DOI:** 10.3389/fphar.2020.476165

**Published:** 2020-09-08

**Authors:** Jingyi Huang, Zhichao Wang, Han Xue, Ailing Cao, Cassidy Turner, Jing Wang, Li Zhang, Jinghai Wang, Na Xiao, Jie Xu, Xianmei Zhou, Hailang He

**Affiliations:** ^1^ Department of Respiratory Medicine, Affiliated Hospital of Nanjing University of Chinese Medicine, Nanjing, China; ^2^ Department of Respiratory Medicine, Jiangsu Province Hospital of Chinese Medicine, Nanjing, China; ^3^ Arizona Metabolomics Laboratory, College of Health Solutions, Arizona State University, Scottsdale, AZ, United States

**Keywords:** Huisheng oral solution, chemotherapy, non–small cell lung cancer, systematic review, meta-analysis, effectiveness

## Abstract

**Background:**

Platinum-based chemotherapy is one of the first line therapies for the advanced non–small cell lung cancer (NSCLC), even though its high toxicity and limited clinical effects cannot be neglected. Huisheng oral solution (HSOS) has been widely used as an adjuvant chemotherapy drug for NSCLC in China. To systematically analyze the therapeutic effects of the combination of HSOS and platinum-based chemotherapy, a comprehensive meta-analysis was performed.

**Objectives:**

This systematic review and meta-analysis aimed to evaluate the effectiveness and safety of HSOS for NSCLC.

**Methods:**

The randomized controlled trials (RCTs) were selected on seven medical databases up to June 2020, including advanced NSCLC treatment using HSOS plus platinum-based chemotherapy versus chemotherapy alone. We followed the PRISMA checklist in general, applying Cochrane handbook 5.1.0, GRADE Pro GDT, RevMan 5.3, Stata12.0, and TSA 0.9.5.10 Beta to evaluate the quality of the study and analyze the data.

**Results:**

Based on Cochrane handbook 5.1.0, 15 RCTs consisting 1165 patients met the criteria and were selected for further analysis. Compared to chemotherapy alone, the chemotherapy combined with HSOS significantly improved objective tumor response (ORR) [RR = 1.38, 95% CI (1.19, 1.59), *P* < 0.0001], disease control rate (DCR) [RR = 1.10, 95% CI (1.04, 1.16), *P* = 0.0006], and Karnofsky performance status (KPS) [RR = 1.79, 95% CI (1.41, 2.26), *P* < 0.00001]. However, there was no evidence of improvement in the 1-year survival rate [RR = 1.37, 95% CI (0.98, 1.92), *P* = 0.07]. In terms of the side effects, HSOS administered concurrently with chemotherapy resulted in a serial of substantial benefits: lower toxicity to white blood cell [RR = 0.30, 95% CI (0.20, 0.43), *P* < 0.00001], lower platelet toxicity [RR = 0.56, 95% CI (0.34, 0.92), *P* = 0.02], and reduced incidence of vomiting [RR = 0.52, 95% CI (0.29, 0.92), *P* = 0.03].

**Conclusions:**

The meta-analysis indicated that HSOS plus platinum-based chemotherapy was more beneficial for patients, as the therapy could synergize antitumor activity and could attenuate toxicity. The finding requires confirmation by further rigorously designed RCTs.

## Introduction

Lung cancer is a leading cause of cancer death for both males and females (Bray et al., 2018). Non–small cell lung cancer (NSCLC) accounts for 80−85% of all the lung cancer cases (Health Commission Of PRC, 2019).

For early-stage NSCLC patients, surgery is the most effective treatment and remains the first choice of action. Unfortunately, by the time NSCLC patients received their first diagnosis, more than half of the patients are already in an advanced stage and therefore lose the opportunity to receive surgery (Wang et al., 2019). Hence, other treatments, such as systemic chemotherapy, radiotherapy, targeted therapy, and immunotherapy become doctors’ choices. According to the National Comprehensive Cancer (NCCN) Guidelines, platinum-based chemotherapy is the dominant treatment for the advanced NSCLC due to its effectiveness in reducing tumor size (Ettinger et al., 2019); however, it is also associated with inadequate effectiveness and some side effects (some patients cannot receive the chemotherapy treatment any further due to the serious side effects). Therefore, it is extremely essential to seek optional treatment approaches to improve the clinical effectiveness and to reduce side effects.

As a form of complementary and alternative approach, traditional Chinese medicine (TCM) has been commonly used for lung cancer, which shows potential benefits in improving the therapeutic effectiveness and reducing the toxicity of chemotherapy (McCulloch et al., 2006; Li et al., 2013; Cao et al., 2017; Wang et al., 2019). Huisheng oral solution (HSOS), an oral liquid formulation, is composed of extracts of 34 Chinese herbal medicines, including ginseng, cyperi rhizoma, angelica, leonuri herba, sparganii rhizoma, *Trogopterus* *dung*, carapax trionycis, olibanum, carthami flos, chuanxiong rhizoma, *persicae* semen, rhei radix, hirudo, caryophylli flos, and ferulae resina, etc. As an adjuvant chemotherapy drug, HSOS has been approved by the China Food and Drug Administration for the treatment of lung and liver cancer. It has been widely used for cancer treatment in China for a long time; some unique biological effects have also been reported using HSOS in animal/human. Wei and co-workers have discovered that HSOS can reduce the incidence of pulmonary thromboembolism and metastasis in mice bearing Lewis lung carcinoma, and the potential mechanisms are associated with ameliorating a blood hypercoagulable state, decreasing tumor angiogenesis, and enhancing immunity (Wang et al., 2014). In another study, it was demonstrated that HSOS can significantly reduce blood hypercoagulability, microthrombosis, and secondary fibrinolysis in a rat model of thrombosis (Liu S. Q. et al., 2013). A Chinese expert consensus has recommended that HSOS could be applied to perioperative anticoagulation therapy of lung cancer patients after surgery (Zhou et al., 2016).

Several studies have shown that HSOS could markedly improve the quality of life and survival rate of lung cancer patients (Xing, 2011; Li et al., 2016). The latest meta-analysis (Li et al., 2016) demonstrated that HSOS combined with chemotherapy could increase the tumor response, thus improve the quality of life, and decrease the risk of the white blood cells (WBCs) toxicity and gastrointestinal adverse effects compared with chemotherapy alone in patients with NSCLC. However, only ten randomized controlled trials (RCTs) with 711 patients were included in the previous meta-analysis, and the quality of enrolled trials was not satisfactory. Recently, there are some new clinical studies evaluating the effectiveness of HSOS combined with systematic chemotherapy for NSCLC (Jia et al., 2013; Wei et al., 2014; Zeng and Zhang, 2014; Du et al., 2015; Huang, 2015; Yang, 2018). Therefore, we conducted this updated systematic review and meta-analysis following PRISMA checklist in general to comprehensively evaluate all related studies with an expectation to provide stronger evidence for the clinical application of HSOS for lung cancer (**Supplementary Material Table 1**).

## Methods

### Literature Search Strategy

A comprehensive literature search was performed in the following electronic databases through June 2020: PubMed, EMBASE, the Cochrane Library, China National Knowledge Infrastructure Database (CNKI), WanFang Database, VIP Database for Chinese Technical Periodicals (VIP), and China Biological Medicine Database (CBM). Medical subject headings (MeSH) and free text words were combined to retrieve all of the potentially related studies. Two reviewers (Jingyi Huang and Han Xue) have conducted the literature search independently with the search strategy (Neoplasm [Mesh] OR Lung Neoplasm [Mesh] OR Pulmonary Neoplasms OR Pulmonary Neoplasm OR Lung Cancer OR Thoracic Neoplasm OR Pulmonary Cancer OR Lung Carcinoma OR Pulmonary Carcinoma OR NSCLC OR Non-small Cell Lung Cancer) AND (Huisheng OR Huisheng Oral Solution OR Huisheng Oral Liquid OR Huisheng Koufuye)).

### Inclusion Criteria

According to the epidemiological data and research status mentioned above, we believed that the research included in the meta-analysis should meet the following criteria: (1) Patients were diagnosed as advanced NSCLC by histopathological or cytological diagnostic criteria. (2) The studies provided the treatment group with HSOS in combination with chemotherapy and the control group with chemotherapy alone. (3) The study design was confined to RCT. (4) The outcome should include at least one of the following indicators: ① Objective tumor response (ORR), ② disease control rate (DCR), ③ Karnofsky performance score (KPS), ④ 1-year survival rate, ⑤ WBC toxicity, ⑥ platelet toxicity, and ⑦ vomiting toxicity. The data should have sufficient details to ensure the calculation of the risk ratios and its 95% CIs for each outcome.

### Exclusion Criteria

Relevant clinical trials were manually removed if any of the following factors was identified: (1) duplicated articles, (2) nonrandomized controlled trial, (3) not being advanced NSCLC, (4) inappropriate interventions, (5) incomplete data, and (6) irrelevant to outcome indicators.

### Outcome Measures

The main indicators include ORR and DCR, and secondary indicators include KPS, 1-year survival rate, and WBC toxicity, platelet toxicity, and vomiting toxicity. According to the Response Evaluation Criteria in Solid Tumors (RECST) developed by the World Health Organization (WHO), the tumor responses were divided into complete response (CR), partial response (PR), stable disease (SD), and progressive disease (PD). The ORR and DCR were defined as CR + PR and CR + PR + SD, respectively. KPS was employed to investigate the performance of patients using a 10-point change as the cut-off for improvement or deterioration, and the improved performance status was calculated as the number of patients with improved KPS (>10-point increase) divided by the total. One-year survival rate refers to the proportion of tumor patients surviving for more than 1 year after a variety of comprehensive treatments. According to the 5-point WHO scale, the reduction of severe chemotherapy toxicity was calculated as the number of patients with any severe toxicity (WHO grade 3 or 4) divided by the total.

### Data Extraction and Quality Assessment

Two review authors (JH and HX) have independently searched and screened the articles and extracted and examined all the data. The data extracted included: (1) basic information such as language, year of publication, and name of the first author; (2) number of participants, physical status, and TNM stage information in each group; and (3) details of interventions and outcomes from each study. The two researchers (JH and HX) have also evaluated the methodological quality of all the RCTs based on the criteria in the Cochrane evaluation handbook of RCTs 5.1.0. Random sequence generation, allocation concealment, blinding (or masking), incomplete data assessment, selective outcome reporting, and other sources of bias were assessed with three potential responses: yes, no, and unclear. At the end of the study, the two investigators used GRADE Pro GDT Online software to evaluate the reliability of the evidence related to each outcome, according to Grading of Recommendations Assessment, Development and Evaluation (GRADE) standard. Any disagreements were resolved through consultation or by consultation with the two other reviewers (HH and XZ).

### Statistical Analysis

According to the RevMan 5.3 software (Cochrane Collaboration), the weighted mean differences (WMD) and relative risk (RR) with 95% confidence were calculated to compare continuous and dichotomous variables, respectively. If a low heterogeneity in pooled studies was observed (*I^2^* ≤ 50%), then a fixed effect model would be adopted. Conversely, the random effects model was applied due to significant heterogeneity (*I^2^* > 50%). If more than 10 studies were included for a meta-analysis, publication bias would be evaluated via funnel plots, and Egger’s test was further carried out to assess funnel plot asymmetry with STATA 12.0 software (He et al., 2013). A sensitivity analysis of ORR and DCR was performed by deleting each study in sequence and re-conducting meta-analysis of the remaining studies. In order to examine the strength of the evidence and whether more randomized controlled trials are needed for sufficient conclusion regarding mortality benefit, we performed the trial sequential analysis (TSA) to guard against false positive (type I) or false negative (type II) errors. TSA software, Copenhagen Trial Unit, version 0.9.5.10 Beta was used to conduct the analysis.

## Results

### Study Identification

**Figure 1:**
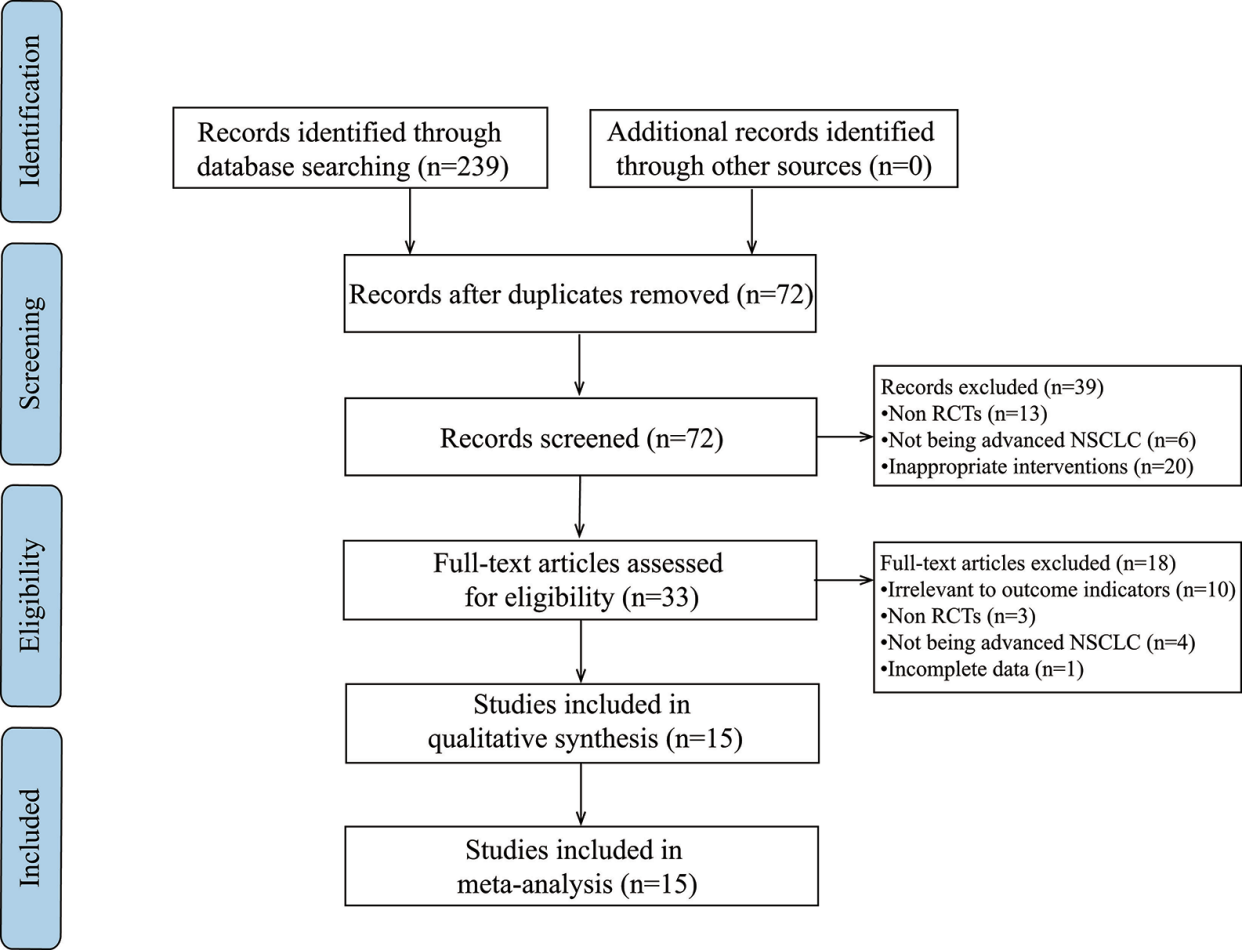
PRISMA flow diagram of the literature search process.

Based on the search strategy, 239 potentially relevant studies were identified. After applying the exclusion criteria, 15 articles were determined for further analysis (**Figure 1**).

### Characteristics of Included Studies

15 RCTs including 1,165 patients were included in the present meta-analysis (Wang et al., 2007; Fang et al., 2009; Ding et al., 2011; Liu et al., 2011; Xu et al., 2011; Yu et al., 2011; Sun, 2012; Jia et al., 2013; Liu X. et al., 2013; Zhang, 2013; Wei et al., 2014; Zeng and Zhang, 2014; Du et al., 2015; Huang, 2015; Yang, 2018). **Table 1** lists all the baseline features of the eligible studies, including the author, the year of publication, the number of cases, the performance status, the TNM stage, the details of intervention, and the results. All of the studies were conducted in China and the year of publication is between 2007 and 2018. The total number of cases was 594 from experimental group and 571 from control group. All the mentioned performance status had KPS ≥ 6, PS ≤ 1, and the stages of NSCLC TNM were III-IV. The control group was given the first-line platinum-based chemotherapy regimen and supportive therapy (including COD, CAP, MFD, TP, NP, GP, and DP). In addition to the first-line chemotherapy regimen containing platinum, the experimental group was supplemented with a standardized regimen of HSOS (t.i.d, po, 30 mL per dose, at least 14 days of a continuous use). The detailed information of HSOS are listed in **Table 2**. The clinical effectiveness was evaluated by ORR, DCR, KPS, and 1-year survival rate, while the toxicity was evaluated by WBC toxicity, PLT toxicity, and vomiting reaction.

**Table 1:** Baseline characteristics of included studies.

Study	No.	Stage	Gender (M/F)		Age (Y)		Interventions		Dose of chemotherapy (mg/m^2^)	Dose of HSOS	Duration (weeks)	Outcomes	
	T/C		T	C	T	C	T	C			T/C		
Yang, 2018	34/34	III-IV	19/15	18/16	42–76(61)	42–73(62)	TP+HSOS	TP	T:150 + DDP:75	30 mL/d	6/6		①②
Du et al., 2015	42/34	IIIb-IV	26/16	19/14	38–79(62)	36–76(57)	PBC + HSOS	PBC	NR	30 mL/d	6/6		③
Huang, 2015	75/80	III-IV	90/65		35–79(57.4)		COD/CAP/MFD + HSOS	COD/CAP/MFD	NR	30 mL/d	12/12		①②④
Wei et al., 2014	33/33	IIIb-IV	45/21		NR		TP + HSOS	TP	T:175 + DDP:75	30 mL/d	6/6		①②③
Zeng and Zhang, 2014	56/54	III-IV	83/27		52–68(60.4)		NP + HSOS	NP	NR	30 mL/d	6/6		③
Jia et al., 2013	42/40	IIIb-IV	23/17	26/16	39–72		43–70	NP + HSOS	NP	N:25 + DDP:75	30 mL/d	6/6		①
Liu X. et al., 2013	37/30	IIIb-IV	21/16	22/8	55.5	56	NP + HSOS	NP	N:25 + DDP:75	30 mL/d	6/6		①②⑤⑥
Zhang, 2013	31/31	III-IV	37/15		35–70(52.5)		TP + HSOS	TP	T:150 + DDP:70	30 mL/d	6/6		①②③
Sun, 2012	18/17	IIIa-IV	21/14		41–77(57±10)		CAP/GP + HSOS	CAP/GP	CTX:400 + ADM:50 + DDP:25; G:1250 + DDP:25	30 mL/d	6/6		①②④
Ding et al., 2011	35/32	III-IV	41/26		35–78(56.5)		NP + HSOS	NP	N:25 + DDP:25	30 mL/d	3/3		①②③⑤⑥⑦
Liu et al., 2011	32/35	III-IV	46/21		42–70		TP + HSOS	TP	T:80 + DDP:80	30 mL/d	6/6		①②
Xu et al., 2011	35/34	III-IV	48/21		42–73		GP/TP + HSOS	GP/TP	T:75 + DDP:25 OR G:1250 + DDP:25	30 mL/d	6/6		①②⑤⑥⑦
Yu et al., 2011	30/30	III-IV	40/20		32–70		NP + HSOS	NP	N:25 + DDP:75	30 mL/d	6/6		①②③⑤
Fang et al., 2009	44/42	IIIb-IV	26/18	25/17	38–77(58)	35–75(57.4)	TP + HSOS	TP	T:75 + DDP:80	30 mL/d	6/6		⑤⑥
Wang et al., 2007	50/45	III-IV	32/18	30/15	35-65(55)	38-70(56)	NP + HSOS	NP	N:25 + C:300/DDP:80-100	30 mL/d	6/6		①②③⑤⑥

No., number of participants; T, treatment; C, control; NR, not reported; M, male; F, female; Y, year; N, navelbine; G, gemcitabine; T, taxol; DDP, cisplation; N, navelbine; CTX, Cytoxan; ADM, doxorubicin; C, Carboplatin; PBC, platinum-based chemotherapy; COD, cytoxan + vincristine + platinum; CAP, cytoxan + doxorubicin + platinum; MFD, mitomycin + 5-fluorouracil + platinum; TP, taxol + platinum; NP, navelbine + platinum; GP, gemcitabine + platinum; DP, docetaxel + platinum; ① ORR; ② DCR; ③ KPS; ④ 1-year survival rate; ⑤ white blood cell toxicity; ⑥ platelet toxicity; ⑦ vomiting toxicity.

**Table 2:** The detailed information of HSOS.

Source	Composition	Percentage (%)	Quantitative determination of chemical constituents
Chengdu Di’ao Tianfu Pharmaceutical Group co., Ltd.	Carapax Trionycis	15.34%	HPLC were used for quality control of HSOS. The content of the dominant active compounds including emodin, chrysophanol, eugenol and stachydrine hydrochloride was 0.0113mg/ml, 0.0404mg/ml, 0.7432mg/ml and 0.0945mg/ml respectively.
	*Leonurus japonicus* Houtt.	7.36%	
	*Rheum palmatum* L.	7.36%	
	*Panax ginseng* C.A.Mey.	5.52%	
	*Angelica sinensis* (Oliv.) Diels	3.68%	
	*Paeonia lactiflora* Pall.	3.68%	
	*Rehmannia glutinosa* (Gaertn.) DC.	3.68%	
	*Caesalpinia sappan* L.	2.76%	
	*Foeniculum vulgare* Mill.	2.76%	
	*Prunus armeniaca* L.	2.76%	
	*Prunus persica* (L.) Batsch	2.76%	
	*Syringa oblata* Lindl.	2.76%	
	*Alpinia officinarum* Hance	1.84%	
	*Artemisia argyi* H.Lév. & Vaniot	1.84%	
	Asafoetida	1.84%	
	Boswellia sacra Flueck.	1.84%	
	*Carthamus tinctorius* L.	1.84%	
	*Cinnamomum cassia* (L.) J.Presl	1.84%	
	*Commiphora myrrha* (T.Nees) Engl.	1.84%	
	Conioselinum anthriscoides 'Chuanxiong'	1.84%	
	*Corydalis yanhusuo* (Y.H.Chou & Chun C.Hsu) W.T.Wang ex Z.Y.Su & C.Y.Wu	1.84%	
	*Curcuma longa* L.	1.84%	
	*Cyperus rotundus* L.	1.84%	
	*Dalbergia odorifera* T.C.Chen	1.84%	
	*Iris domestica* (L.) Goldblatt & Mabb.	1.84%	
	Leeches	1.84%	
	*Perilla frutescens* (L.) Britton	1.84%	
	Radde Anemone Rhizome	1.84%	
	*Sparganium stoloniferum* (Buch.-Ham. ex Graebn.) Buch.-Ham. ex Juz.	1.84%	
	*Tabanus*	1.84%	
	*Tetradium ruticarpum* (A.Juss.) T.G.Hartley	1.84%	
	Trogopterus Dung	1.84%	
	*Zanthoxylum armatum* DC.	1.84%	
	*Typha angustifolia* L.	0.92%	

### Methodological Bias of the Included Studies

**Figure 2:**
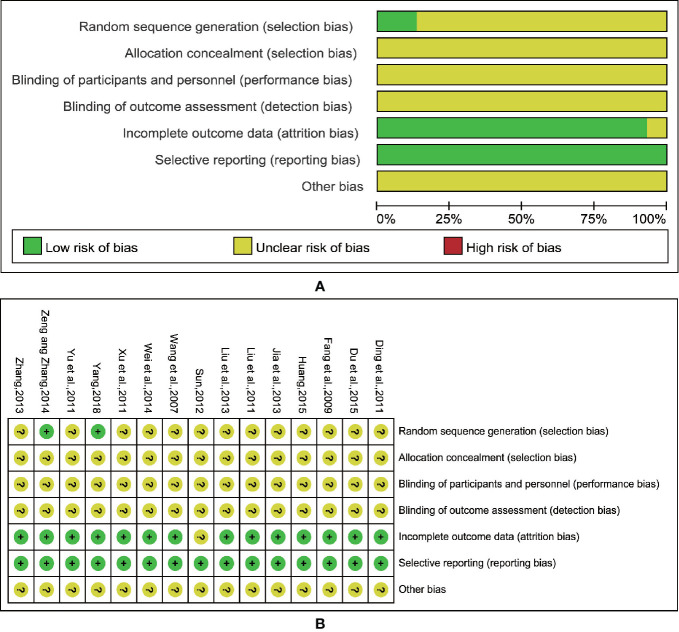
Risk of methodological bias of the included studies. **(A)** Risk of bias graph. **(B)** Risk of bias summary.

All of the included trials mentioned applying a randomization methodology, but only two studies (Zeng and Zhang, 2014; Yang, 2018) specified the method, which was randomized by using random number tables to generate a sequence. We attempted a verification by contacting the authors of the original papers via phone and e-mail, and although extensive effort has been made, we failed to obtain any additional details. None of the trials specified the methods of allocation concealment and the blinding procedures. This indicated that there were unclear risks of bias, including selection bias, performance bias, and detection bias. Twelve trials (Wang et al., 2007; Fang et al., 2009; Ding et al., 2011; Liu et al., 2011; Yu et al., 2011; Jia et al., 2013; Liu X. et al., 2013; Zhang, 2013; Wei et al., 2014; Zeng and Zhang, 2014; Huang, 2015; Yang, 2018) exhibited complete outcome data, and three trials reported drop-out data (Xu et al., 2011; Sun, 2012; Du et al., 2015), two of which used ITT analysis (Xu et al., 2011; Du et al., 2015), and the other one (Sun, 2012) only mentioned the number of exits without explanation of reasons and treatment methods. No trials mentioned selective reporting. And other biases were unclear. The detailed information about the quality of the research methods is shown in **Figure 2**.

### Outcome Measures

#### Objective Tumor Response

**Figure 3:**
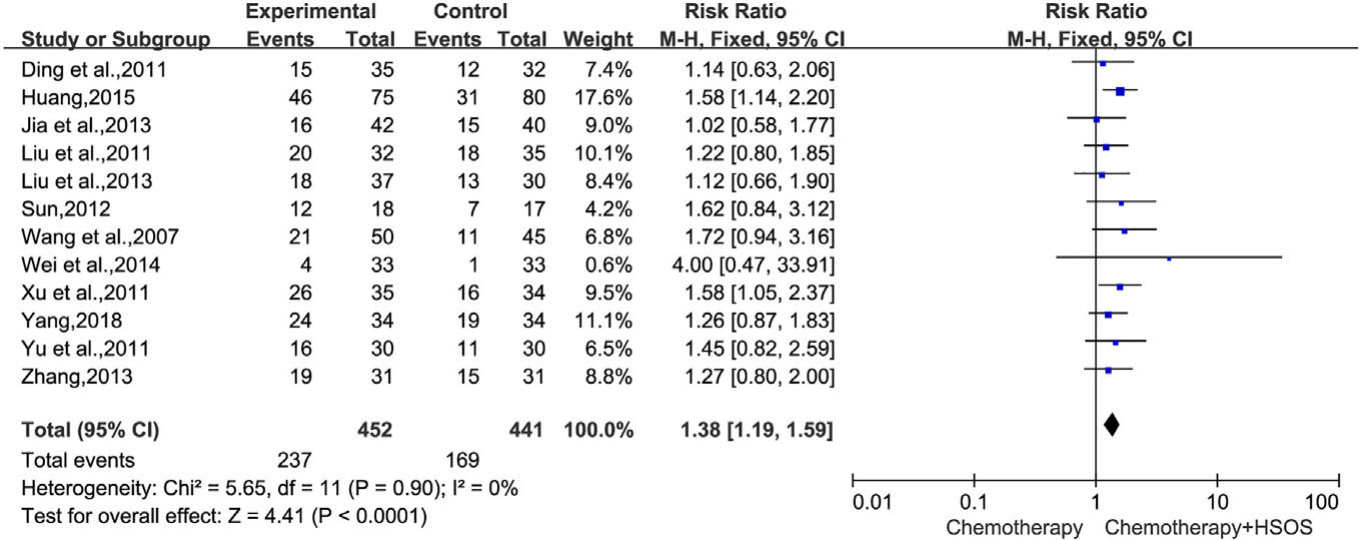
Forest plot of improved ORR with chemotherapy combined with HSOS versus chemotherapy alone.

Twelve trials (Wang et al., 2007; Ding et al., 2011; Liu et al., 2011; Xu et al., 2011; Yu et al., 2011; Sun, 2012; Jia et al., 2013; Liu X. et al., 2013; Zhang, 2013; Wei et al., 2014; Huang, 2015; Yang, 2018), including 893 cases reported ORR (**Figure 3**), had no significant heterogeneity (*I^2^* = 0%, *P* = 0.90). We applied the fixed-effects model for the analysis. The results indicated that the treatment of platinum-based plus HSOS significantly improved the objective tumor response of patients with NSCLC compared with the chemotherapy alone [RR = 1.38, 95% CI (1.19, 1.59), *P* < 0.0001].

#### Disease Control Rate

**Figure 4:**
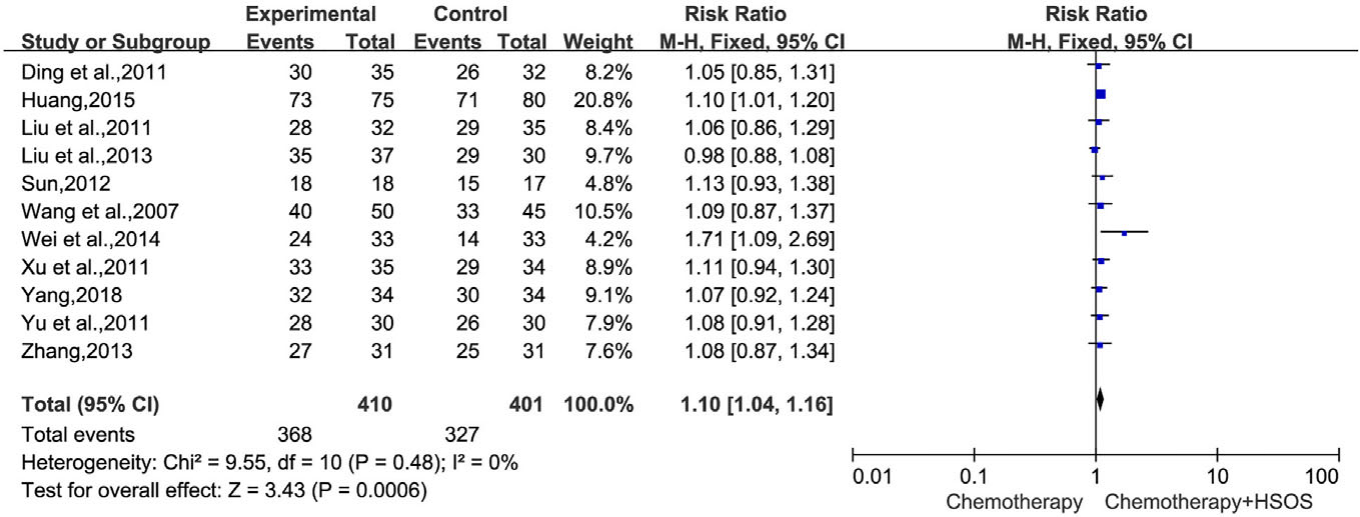
Forest plot of improved DCR with chemotherapy combined with HSOS versus chemotherapy alone.

In 15 trials, 11 trials (Wang et al., 2007; Ding et al., 2011; Liu et al., 2011; Xu et al., 2011; Yu et al., 2011; Sun, 2012; Liu X. et al., 2013; Zhang, 2013; Wei et al., 2014; Huang, 2015; Yang, 2018), including 811 cases, reported DCR (**Figure 4**). The heterogeneity test indicated that the data was homogeneous (*I^2^* = 0%, *P* = 0.48), employing the fixed-effects model in this meta-analysis. In the meta-analysis, a statistically significant difference [RR = 1.10, 95% CI (1.04, 1.16), *P* = 0.0006] existed between HSOS combination group and control group, signifying that a combination of HSOS and chemotherapy could result in a remarkable improvement of DCR.

#### Performance Status

**Figure 5:**
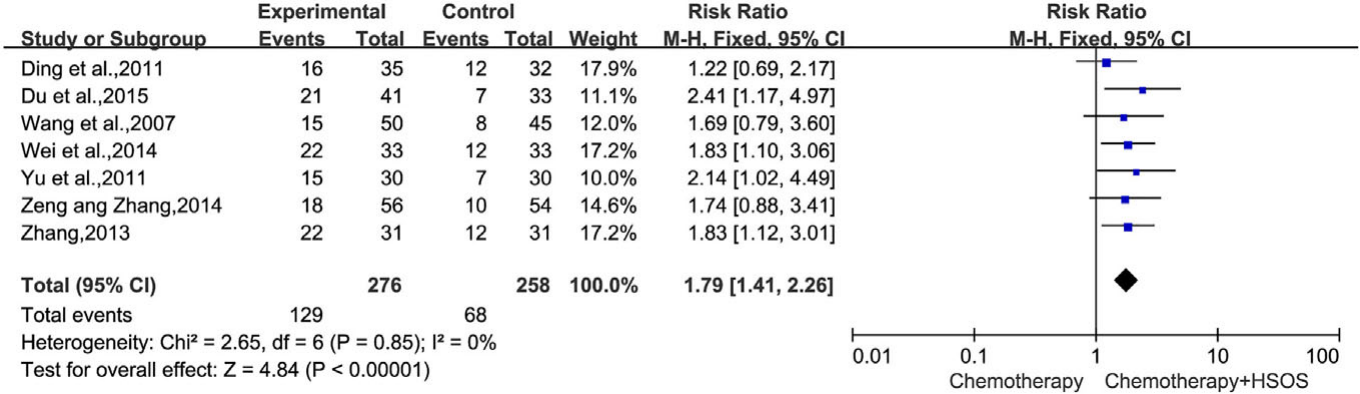
Forest plot of improved KPS with chemotherapy combined with HSOS versus chemotherapy alone.

KPS could be definitively extracted from seven reports (Wang et al., 2007; Ding et al., 2011; Yu et al., 2011; Zhang, 2013; Wei et al., 2014; Zeng and Zhang, 2014; Du et al., 2015). There was no heterogeneity between studies (*I^2^* = 0%, *P* = 0.85). The pooled RR for KPS revealed that there was a better quality of life for the combination treatment of HSOS and chemotherapy, yielding a RR of 1.79 [95% CI (1.41, 2.26), *P* < 0.00001] by fixed-effects model (**Figure 5**).

#### One-Year Survival Rate

**Figure 6:**
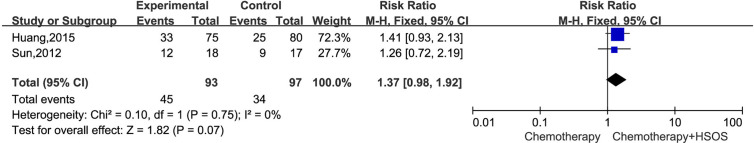
Forest plot of 1-year survival rate with chemotherapy combined with HSOS versus chemotherapy alone.

The incidence of 1-year survival rate was reported in two trials (Sun, 2012; Huang, 2015), which included 190 patients (**Figure 6**). As the heterogeneity test showed (*I^2^* = 0%, *P* = 0.75), a fixed-effects model was applied to calculate the combined RR and 95% CI. The results demonstrated that the HSOS group showed no significant difference in 1-year survival rate compared with control group [RR = 1.37, 95% CI (0.98, 1.92), *P* = 0.07].

#### Reduction in Chemotherapy Toxicity

**Figure 7:**
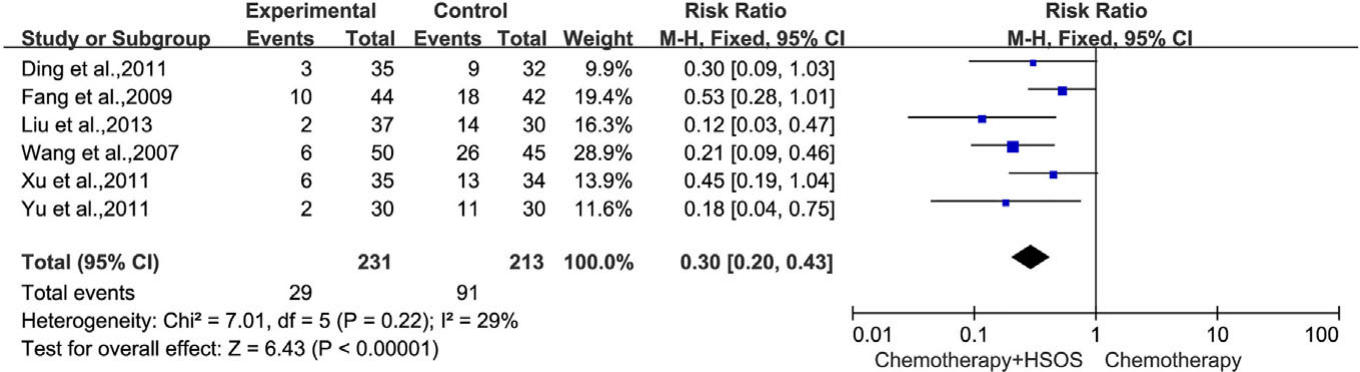
Forest plot of WBC toxicity with chemotherapy combined with HSOS versus chemotherapy alone.

In all studies, six trials (Wang et al., 2007; Fang et al., 2009; Ding et al., 2011; Xu et al., 2011; Yu et al., 2011; Liu X. et al., 2013) including 444 cases reported WBC toxicity (**Figure 7**), which had no significant heterogeneity (*I^2^* = 29%, *P* = 0.22), so a fixed-effects model was applied for analysis. Meta-analysis showed that there was a statistically significant difference between two groups in the toxicity of WBC [RR = 0.30, 95% CI (0.20, 0.43), *P* < 0.00001], indicating that the treatment of Platinum-based chemotherapy plus HSOS had an advantage in alleviating the toxicity of WBC compared with using chemotherapy alone.

**Figure 8:**
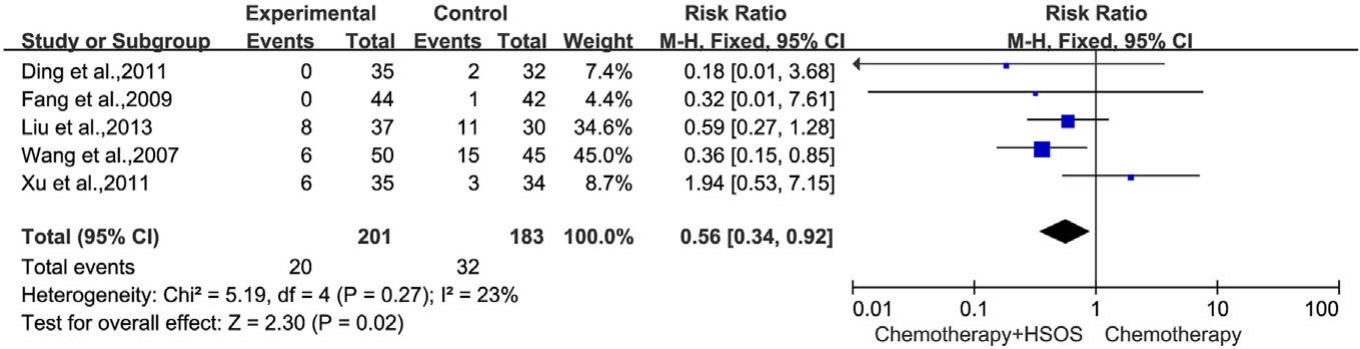
Forest plot of platelet toxicity with chemotherapy combined with HSOS versus chemotherapy alone.

Five studies (Wang et al., 2007; Fang et al., 2009; Ding et al., 2011; Xu et al., 2011; Liu X. et al., 2013) included in this systematic analysis provided the data on platelet toxicity after treatment (**Figure 8**). As there was no statistical heterogeneity (*I^2^* = 23%, *P* = 0.27), the fixed-effects model could be used in this meta-analysis. The results showed that the RR was 0.56 [95% CI (0.34, 0.92), *P* = 0.02], indicating that the application of HSOS exhibited a remarkable reduction in platelet toxicity compared with chemotherapy alone.

**Figure 9:**
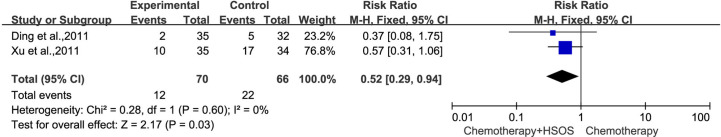
Forest plot of vomiting toxicity with chemotherapy combined with HSOS versus chemotherapy alone.

Two studies (Ding et al., 2011; Xu et al., 2011) showed no significant heterogeneity on vomiting toxicity (*I^2^* = 0%, *P* = 0.60) (**Figure 9**). The results of statistical analysis based on the fixed-effects model demonstrated that the combined treatment can reduce the incidence of vomiting toxicity compared with using chemotherapy alone [RR = 0.52, 95% CI (0.29, 0.92), *P* = 0.03].

### Analysis of Publication Bias

**Figure 10:**
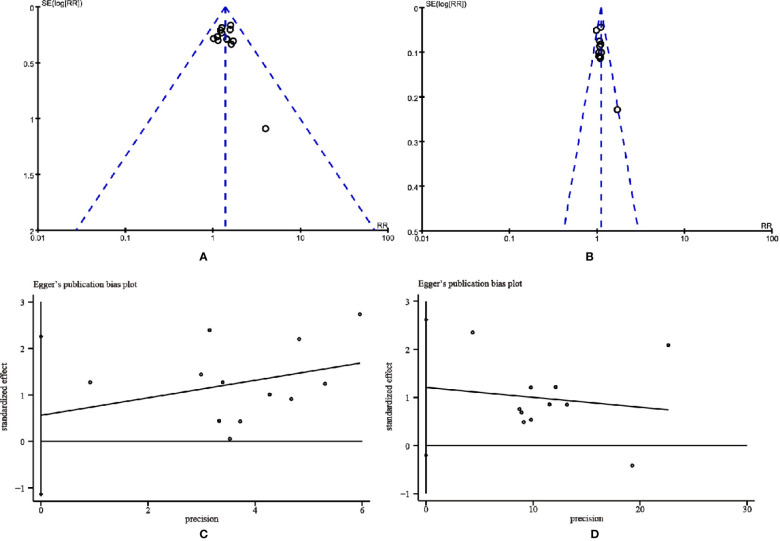
The funnel plots and the Egger’s publication bias plots for assessing publication bias. **(A)** Objective tumor response. **(B)** Disease control rate. **(C)** Objective tumor response. **(D)** Disease control rate.

The publication bias was evaluated by Funnel plots and Egger’s test. The funnel plots showed an asymmetrical distribution in the studies on ORR and DCR, indicating the potential publication bias (**Figures 10A, B**). Thus, Egger’s test was carried out for quantitative analysis. The Egger’s test for ORR (*t* = 0.74, 95% CI, -1.13725 to 2.259829, *P* = 0.478) and DCR (*t* = 1.94, 95% CI, -0.2028584 to 2.619618, *P* = 0.085) revealed that there was no proof of publication bias (**Figures 10C, D**).

### Sensitivity Analysis

**Figure 11:**
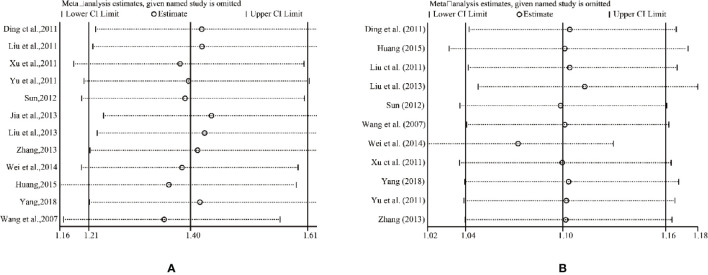
Sensitivity analysis. **(A)** Objective tumor response. **(B)** Disease control rate.

The results of all studies in the fixed-effects model showed good consistency. One trial was deleted each time, and the rest was reanalyzed, resulting in similar results compared with the previous ones. The consistency of the results indicated that the results were authentic and verifiable. For further verification, we implemented a sensitivity analysis of ORR and DCR by STATA 12.0. **Figure 11** indicated that the outcomes were very similar, which had relatively good stability.

### Trial Sequential Analysis

**Figure 12:**
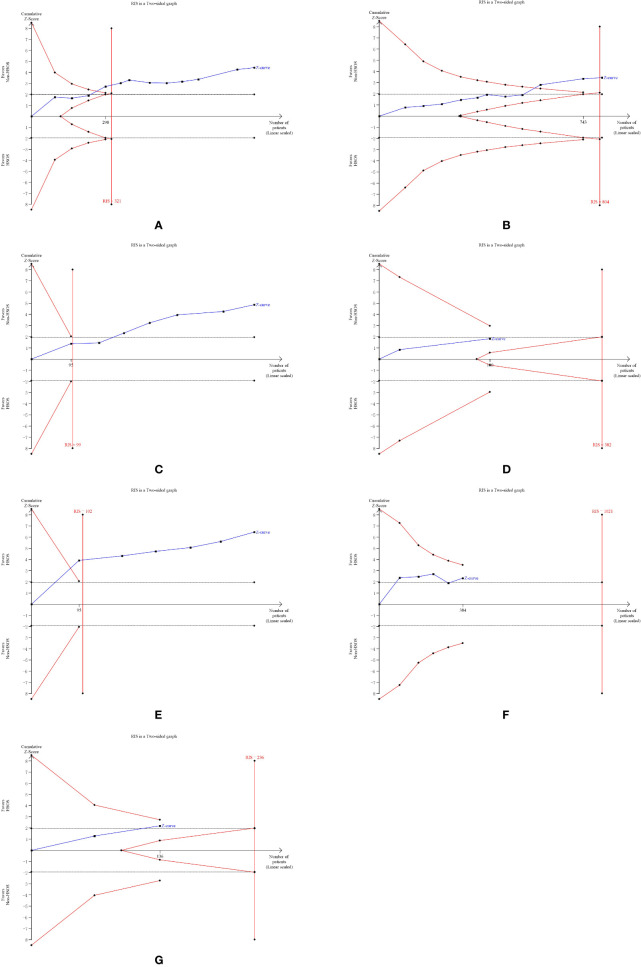
Trial sequential analysis. **(A)** Objective tumor response. **(B)** Disease control rate. **(C)** KPS. **(D)** One-year survival rate. **(E)** White blood cell toxicity. **(F)** Platelet toxicity. **(G)** Vomiting toxicity.

We applied TSA boundaries to assess the robustness of the results and calculate the required information size (RIS) in the meta-analysis. The type I error rate was defined as 5%, the type II error rate was set as 20%, and relative risk reduction (RRR) was derived from the meta-analysis. As shown in **Figure 12A**, the Z-score curve (blue line) crossed the statistical significance boundary (red polylines), the required information size (vertical red line), and the conventional statistical significance boundary corresponding to two-sided p value of 0.05 (dotted black lines). The results indicated that the improvement of ORR in lung cancer patients with HSOS could be considered conclusive with the existing evidence. Also, displayed in **Figures 12B, E**, the improvement in DCR and the reduction in WBC toxicity are definite and well documented. The reduction of vomiting toxicity and platelet toxicity and the improvement of 1-year survival rate need to be demonstrated by more studies (**Figures 12D, F, G**). However, although the graph shown statistical significance, the improvement in KPS may be a false positive result (**Figure 12C**).

### Quality of Evidence

We used GRADE Pro GDT to further evaluate the quality of evidence for the outcomes. Because of some uncertainty about risk of bias, we were moderately confident in outcomes of ORR, DCR, KPS, WBC toxicity, and platelet toxicity. We had very little confidence in the outcomes of vomiting toxicity and 1-year survival rate because, in addition to the possible risk of bias, there were other factors lowered the level of evidence such as insufficient sample size and few relevant studies (**Table 3**).

**Table 3:** Summary of Fingings table.

Outcomes	Anticipated absolute effects^*^ (95% CI)		Relative effect(95% CI)	№ of participants(studies)	Certainty of the evidence(GRADE)
	Risk with non-HSOS	Risk with HSOS			
ORR	383 per 1,000	**529 per 1,000** (456 to 609)	**RR 1.38** (1.19 to 1.59)	893 (12 RCTs)	⨁⨁⨁◯ MODERATE^a^
DCR	815 per 1,000	**897 per 1,000** (848 to 946)	**RR 1.10** (1.04 to 1.16)	811 (11 RCTs)	⨁⨁⨁◯ MODERATE^a^
KPS	264 per 1,000	**472 per 1,000** (372 to 596)	**RR 1.79** (1.41 to 2.26)	534 (7 RCTs)	⨁⨁⨁◯ MODERATE^a^
WBC	427 per 1,000	**128 per 1,000** (85 to 184)	**RR 0.30** (0.20 to 0.43)	444 (6 RCTs)	⨁⨁⨁◯ MODERATE^b^
PLT	175 per 1,000	**98 per 1,000** (59 to 161)	**RR 0.56** (0.34 to 0.92)	384 (5 RCTs)	⨁⨁⨁◯ MODERATE^b^
Vomiting	333 per 1,000	**173 per 1,000** (97 to 313)	**RR 0.52** (0.29 to 0.94)	136 (2 RCTs)	⨁◯◯◯ VERY LOW^b^,^c^,^d^
1-year survival rate	351 per 1,000	**480 per 1,000** (344 to 673)	**RR 1.37** (0.98 to 1.92)	190 (2 RCTs)	⨁◯◯◯ VERY LOW^b^,^c^,^d^

**The risk in the intervention group** (and its 95% confidence interval) is based on the assumed risk in the comparison group and the **relative effect** of the intervention (and its 95% CI); CI, confidence interval; RR, risk ratio.

a All trials mentioned applying a randomization methodology, but only one study specified the method. None of the trials specified the methods of allocation concealment and the blinding procedures.

b All trials mentioned applying a randomization methodology, but none of the studies specified the method. None of the trials specified methods of allocation concealment and the blinding procedures.

c The total sample size did not reach the optimal information size (OIS).

d There were few relevant studies.

## Discussion

In the present study, 15 RCTs including 1,165 patients were analyzed, which ensured an adequate sample size for meta-analysis. According to the WHO guidelines, the present meta-analysis revealed that combining chemotherapy with HSOS in the treatment of advanced NSCLC could significantly improve the ORR, DCR, and the quality of life, as well as decrease the toxicity grade of III or IV when compared with the chemotherapy alone. This result was objective and had nothing to do with any key groups, such as healthcare providers, users, and policy makers. Prior to our study, two meta-analyses evaluating the effects of HSOS plus chemotherapy on lung cancer have been published in Chinese (Xing, 2011; Li et al., 2016); however, no definitive conclusions were reached due to the lower methodological quality of RCTs included. Moreover, the latest meta-analysis, which was conducted by Li et al. in 2015, only included 10 studies involving 711 patients. The insufficient sample size may result in weakening of statistical characteristics. Therefore, we conducted a comprehensive search and added several new articles published in recent years, with the expectation of obtaining some statistical advancement and providing updated evidence for the clinical application of combining HSOS and chemotherapy in the treatment of advanced NSCLC.

Although the present meta-analysis demonstrates favorable outcomes, it is too ambitious to make solid conclusions due to several limitations in methodological quality of the current studies. The methodological quality and the data presentation of the majority of trials were variable and often inadequate. Out of 15 trials, only two provided the detailed information on the randomization method and none reported concealment of treatment allocation. Information on blinding was not provided in any of the studies. In addition, three trials mentioned dropout or withdraw, two of which used ITT analysis, and the other one only mentioned the number of exits without explanation of reasons and treatment methods. Although the absence of relevant information does not mean poor implementation, the lack of such information makes it difficult to evaluate the biases of the trials. Moreover, although we had searched all the mainstream databases in Chinese and English, all of the studies selected were published in China, which could lead to ethical bias. What’s more, differences in duration of HSOS treatment and differences in chemotherapy regimens may increase heterogeneity risk. Additionally, all studies included in the present systematic review used an “A + B versus B” design where patients were randomized to receive chemotherapy plus HSOS versus chemotherapy alone, without a rigorous control for the placebo effect. This kind of design is likely to generate false positive results (Ernst and Lee, 2008). The strength of our review is that we used GRADE and TSA to assess the quality of the evidence and the risks of random errors respectively, which provided further robustness to the results and conclusions. GRADE assessment demonstrated that there was moderate evidence in most of the outcomes including ORR, DCR, KPS, WBC toxicity, and platelet toxicity. In addition, TSA suggested a firm conclusion on the effect of HSOS on the ORR. However, GRADE assessment also showed that the quality of the evidence was ‘very low’ for the outcomes of vomiting toxicity and 1-year survival rate, mostly due to the risk of bias. Moreover, the present meta-analysis showed that the chemotherapy combined with HSOS could improve KPS, but TSA did not confirm this result, which might be a false positive result. Therefore, convinced evidence regarding the efficacy of HSOS combined with chemotherapy in lung cancer patients are still lacking, those results could be referenced to guide future study to focus on the use of HSOS in lung cancer patients. In addition, it is critical to improve the methodological quality of RCTs for future studies and more methodologically rigorous researches are needed to confirm or refute the results reported here.

HSOS is a type of extract from traditional Chinese prescription, known as Huazheng Huisheng Dan, which has been used for hundreds of years in clinic. It has been demonstrated that HSOS could inhibit the growth, invasion, and metastasis of tumor cells by inhibiting the secretion of transforming growth factor (TGF)–β, TGF-α, and vascular endothelial growth factor (VEGF), thus improving the microenvironment of tumor growth and influencing the formation of tumor vascellum (Liu X. et al., 2013; Wei et al., 2014). Moreover, studies have shown that HSOS could improve immune responses by affecting interleukin-2 (IL-2), T cells, natural killer (NK) cells, or lymphokine-activated killer (LAK) cells (Zhao et al., 1998; Ma et al., 2005; Liu X. et al., 2013). In addition to the role in antitumor and immunity, HSOS has also shown anticoagulant and antithrombotic activity. Based on traditional Chinese medicine theory, “promoting blood circulation and removing blood stasis” was an important method for treating cancers. HSOS contains a large proportion of Chinese herbals that can promote blood circulation and remove blood stasis, such as hirudo, gadfly tabanus, and cartham flos, which have all been found to exhibit significant anticoagulation activity (Zhou et al., 2016). Blood hypercoagulation is widely considered as highly relevant to cancer, as hypercoagulation will lead to the processes of tumor growth, progression, and metastasis (Mandoj et al., 2019). HSOS is thus a potential treatment for cancer patients, as they often exhibit hypercoagulation, one of the most common causes of cancer-related death (Lee et al., 2017). What’s more, HSOS is of good safety profile and does not increase the risk of bleeding (Yang et al., 2017). However, to clarify the HSOS function as an adjunct to chemotherapy, future studies should be focused on the specific mechanisms.

## Conclusion

In conclusion, evidence was found to support the fact that combination of HSOS with platinum-based chemotherapy may increase advanced NSCLC treatment effectiveness and reduce its systemic toxicity. To further support the conclusion, larger sample size and well-designed RCTs are warranted.

## Data Availability Statement

All datasets generated for this study are included in the article/**Supplementary Material**.

## Author Contributions

XZ and HH contributed conception and design of the study. JH searched the database, evaluated studies for inclusion, extracted data, and then cross-checked with HX. AC, LZ, NX, and JX assisted in literature retrieval and data analysis. JH and HH wrote the first draft of the manuscript. HX, AC, CT, JW, and JHW wrote sections of the manuscript. ZW completed the final version. XZ and HH were responsible for quality control of the study. All authors contributed to the article and approved the submitted version.

## Funding

This work was supported by the Project of Key Discipline for TCM Construction of Jiangsu Province, China (no. JS1302).

## Conflict of Interest

The authors declare that the research was conducted in the absence of any commercial or financial relationships that could be construed as a potential conflict of interest.

## References

[B1] Bray F. Ferlay J. Soerjomataram I. Siegel R. L. Torre L. A. Jemal A. (2018). Global cancer statistics 2018. CA Cancer J. Clin. 68 (6), 394–424. 10.3322/caac.21492 30207593

[B2] Cao A. He H. Jing M. Yu B. Zhou X. (2017). Shenfu injection adjunct with Platinum-Based chemotherapy for the treatment of advanced Non-Small-Cell lung cancer: A Meta-Analysis and systematic review. Evid. Based Complement Alternat. Med. 2017, 1068751. 10.1155/2017/1068751 29234363 PMC5688370

[B3] Ding N. Song Y. Zhang F. (2011). Clinical observation of 35 cases of mid- advanced NSCLC with the adjuvant therapy of Huisheng oral liquid. Shandong Med. J. 51, 118–119. 10.3969/j.issn.1002-266X.2011.51.073

[B4] Du J. Yin Y. Qiao H. (2015). Effect of maintenance therapy with Huisheng Oral Liquid for patients in mid-advanced non-small cell lung cancer. Clin. Focus 30, 641–644. 10.3969/j.issn.1004-583X.2015.06.011

[B5] Ernst E. Lee M. S. (2008). A trial design that generates only ‘‘positive’’ results. J. Postgrad. Med. 54, 214–216. 10.4103/0022-3859.41806 18626172

[B6] Ettinger D. S. Wood D. E. Aisner D. L. Akerley W. Bauman J. (2019). NCCN Guidelines Version 4.2019 Non-Small Cell Lung Cancer. Available at: https://www.nccn.org/professionals/physician_gls/pdf/nscl.pdf (Accessed May 08 2019).

[B7] Fang X. Guan M. Liu G. Mao H. (2009). Prevention of arrest of bone marrow by chemotherapy in patients with non-small cell lung cancer treated by Huisheng oral liquid. J. China Trad. Chin. Med. Inf. 1, 40–41.

[B8] He H. Zhou X. Wang Q. Zhao Y. (2013). Does the couse of astragalus-containing chinese herbal prescriptions and radiotherapy benefit to non-small-cell lung cancer treatment: A meta-analysis of randomized trials. Evid. Based Complement Alternat. Med. 2013, 426207. 10.1155/2013/426207 24454494 PMC3878281

[B9] Health Commission Of PRC, N (2019). Chinese guidelines for diagnosis and treatment of primary lung cancer 2018 (English version). Chin. J. Cancer Res. 31, 1–28. 10.21147/j.issn.1000-9604.2019.01.01 30996564 PMC6433582

[B10] Huang X. (2015). Clinical observation on treating mid-advanced lung cancer with Huisheng oral liquid combined with chemotherapy. J. Baotou Med. Coll. 31 39, 40.

[B11] Jia Y. Wei Y. Shen H. Tian Y. Zang A. (2013). Effects of hui-sheng oral liquid on the expression of interleukin-6 and interleukin-18 of patients with advanced non-small-cell lung cancer. Anti-Tumor Pharm. 3, 447–450. 10.3969/j.issn.2095-1264.2013.109

[B12] Lee M. J. Chung J. W. Ahn M. J. Kim S. Seok J. M. (2017). Hypercoagulability and mortality of patients with 375stroke and active cancer: The OASIS-CANCER study. J. Stroke 19, 77–87. 10.5853/jos.2016.00570 28030894 PMC5307941

[B13] Li S. G. Chen H. Y. Ou-Yang C. S. Wang X. X. Yang Z. J. Tong Y. (2013). The efficacy of Chinese herbal medicine as an adjunctive therapy for advanced non-small cell lung cancer: A systematic review and meta-analysis. PloS One 8, e57604. 10.1371/journal.pone.0057604 23469033 PMC3585199

[B14] Li L. Chen J. Zhang D. Guo Y. (2016). Clinical efficacy of huisheng oral liquid combined with chemotherapy in the treatment of lung cancer: A meta analysis. Drug Economy 004, 16–19. 10.12010/j.issn.1673-5846.2016.04.004

[B15] Liu Y. Wei H. Dai X. Hou Z. Liu X. (2011). Clinical observation of 32 cases of mid-advanced non-small cell lung cancer treated by Huisheng oral liquid combined with TP chemotherapy. Shandong Med. J. 51, 106. 10.3969/j.issn.1002-266X.2011.51.065

[B16] Liu S. -Q. Guo J. Y. Du J. Deng Q. He Z. -J. Lin H. -Y (2013). Anticoagulant effect of Huisheng Oral Solution in a rat model of thrombosis. Indian J. Pharmacol. 45, 359–364. 10.4103/0253-7613.115018 24014911 PMC3757604

[B17] Liu X. Liu Y. Dai X. Hou Z. (2013). The clinical significance of NP adjuvant anticancer Chinese Huisheng oral liquid in treatment of non-small cell lung cancer. Clin. Focus 28, 810–811. 10.3969/j.issn.1004-583X.2013.07.033

[B18] Ma D. Lin P. Deng G. (2005). Influence on Hui-sheng oral liquid to Cellular Immunity Function of Aged Cancer 390. Patients after Chemotherapy. J. Chengdu University of TCM. 52–54.

[B19] Mandoj C. Tomao L. Conti L. (2019). Coagulation in brain tumors: Biological basis and clinical implications. Front. Neurol. 392 (10), 181. 10.3389/fneur.2019.00181 PMC6436068 30949114

[B20] McCulloch M. See C. Shu X. J. Broffman M. Kramer A. Fan W. (2006). Astragalus-based Chinese herbs and platinum-based chemotherapy for advanced non-small-cell lung cancer: Meta-analysis of randomized trials. J. Clin. Oncol. 24, 419–430. 10.1200/JCO.2005.03.6392 16421421

[B21] Sun X. (2012). Huisheng oral liquid combined with chemotherapy in the treatment of advanced lung cancer. Chin. J. Pract. Med. 39, 107–108. 10.3760/cma.j.issn.1674-4756.2012.03.051

[B22] Wang D. Yan P. Mo Z. Li Z. (2007). Effect of Huisheng oral liquid on hemogram and immune function in patients with non-small cell lung cancer undergoing chemotherapy. Chin. J. Inf. On Trad. Chin. Med. 14, 67–78. 10.3969/j.issn.1005-5304.2007.10.038

[B23] Wang W. Wang H. Wang C. M. Gou S. Chen Z. H. Guo J. (2014). Treatment with Huisheng oral solution inhibits the development of pulmonary thromboembolism and metastasis in mice with Lewis lung carcinoma. Oncol. Lett. 7, 87–94. 10.3892/ol.2013.1661 24348827 PMC3861555

[B24] Wang Z. Feng F. Wu Q. Jin Y. Gu C. Xu Y. (2019). Disodium cantharidinate and vitamin b6 injection adjunct with Platinum-Based chemotherapy for the treatment of advanced Non-Small-Cell lung cancer: A Meta-Analysis. Evid. Based Complement Alternat. Med. 2019:9386273. 10.1155/2019/9386273 30992710 PMC6434307

[B25] Wei Y. Duan M. Jia Y. Zang A. (2014). Effect of Hui Sheng oral soluion on the treatment of advanced non-small cell lung cancer and its effect on the expression of TGF-β, -α. Chin. J. Gerontol. 34, 6072–6073. 10.3969/j.issn.1005-9202.2014.21.062

[B26] Xing G. (2011). Meta analysis of the short-term efficacy and quality of life of NSCLC in combination with chemotherapy in the treatment of advanced stage. Shandong Med. J. 51, 111–113. 10.3969/j.issn.1002-266X.2011.51.069

[B27] Xu X. Su J. Fu X. Xue F. Huang Q. (2011). Clinical evaluation of Huishengkoufuye combined with first-line chemotherapy on Treatment in Patients with Midadvanced Non-small Cell Lung Cancer. Shandong Med. J. 51, 80–81. 10.3969/j.issn.1002-266X.2011.04.039

[B28] Yang X. Zhang H. Kong F. Wang G. Gu Q. (2017). Effect of Huisheng oral solution on coagulation function 421in perioperative period in patients with primary lung cancer. J. Thorac. Dis. 9, 1891–1902. 10.21037/jtd.2017.06.6 28839987 PMC5542976

[B29] Yang L. (2018). Therapeutic effect of TP regimen combined with huisheng oral liquid in the treatment of advanced non-small cell lung carcinoma. Med. Inf. 31, 17–19. 10.3969/j.issn.1006-1959.2018.17.005

[B30] Yu Y. Zhang L. Lei J. Xu T. Tai Y. Cao F. (2011). Effect of Huisheng oral liquid on advanced non-small cell lung cancer. Shandong Med. J. 51, 101–102. 10.3969/j.issn.1002-266X.2011.51.062

[B31] Zeng J. Zhang J. (2014). 56 cases of mid-advanced lung cancer treated with Huisheng oral liquid. China Pharm. 23, 73.

[B32] Zhang W. (2013). Clinical efficacy of Huisheng oral liquid in the adjuvant therapy of mid-advanced non-small cell lung cancer. World Latest Med. Inf. 13, 207–208. 10.3969/j.issn.1671-3141.2013.14.158

[B33] Zhao X. Duan D. Zhang A. Wang Z. Zhu M. (1998). Effect of antineoplastic agent Huisheng oral liquid on 429human IL-2 level and activity of LAK cells. World Chinese J. Digestol. 44–46.

[B34] Zhou Q. Liu J. Yang X. Zhang H. Kong F. Wang G. (2016). [Experts Consensus on Huisheng Oral Solution for Lung Cancer Anticoagulation Treatment at Perioperation Period, (2016 version)]. Zhongguo Fei Ai Za Zhi 19, 721–724. 10.3779/j.issn.1009-3419.2016.11.01 27866513 PMC5999641

